# Clinical and radiological outcomes of a cervical cage with integrated fixation

**DOI:** 10.1097/MD.0000000000014097

**Published:** 2019-01-18

**Authors:** Nicolas Lonjon, Emmanuel Favreul, Jean Huppert, Eric Lioret, Manuel Delhaye, Ramzi Mraidi

**Affiliations:** a Department of Neurosurgery, Gui de Chauliac Hospital, Montpellier; b Orthopaedic Surgery, Clinique Saint Charles, Lyon; c Department of Neurosurgery, Clinique du Parc, St-Priest-en-Jarez; d Department of Neurosurgery, University Hospital, Tours; e Department of Neurosurgery, Clinique Saint Léonard, Trélazé; f Clinical Affairs Department, Zimmer Biomet Spine, Troyes, France.

**Keywords:** cervical DDD, discectomy, interbody fusion, low profile system

## Abstract

Cervical cages with integrated fixation have been increasingly used in anterior cervical discectomy and fusion (ACDF) to avoid complications associated with anterior cervical plates. The purpose of this paper is to provide 2-year follow-up results of a prospective study after implantation of a cervical cage with an integrated fixation system.

This was a prospective multicenter outcome study of 90 patients who underwent ACDF with a cage with integrated fixation. Fusion was evaluated from computed tomography images (CT-images) by an independent laboratory at 2-year follow-up (FU). Clinical and radiological findings were recorded preoperatively and at FU visits and complications were reported.

At 24 months, the fusion rate was 93.4%. All average clinical outcomes were significantly improved at 2 years FU compared to baseline: neck disability index (NDI) 18.9% vs 44.4%, visual analog scale (VAS) for arm pain 18.2 mm vs 61.9 mm, VAS for neck pain 23.9 mm vs 55.6 mm. Short form-36 (SF-36) scores were significantly improved. One case of dysphagia, which resolved within 12 months, and 1 reoperation for symptomatic pseudarthrosis were reported. Subsidence with no clinical consequence or reoperation was reported for 5/125 of the implanted cages (4%). There was also 1 case of per-operative vertebral body fracture that did not require additional surgery. Superior and inferior adjacent discs showed no significant change of motion at 2-year FU compared to baseline. Disc height index (DHI) and lordosis were enhanced and these improvements were maintained at 1 year.

The ACDF using cages with an integrated fixation system demonstrated reliable clinical and radiological outcomes and a high interbody fusion rate. This rate is comparable to the rate reported in recent series using other implants with integrated fixation, but the present device had a lower complication rate.

## 1 Introduction

Anterior cervical discectomy and fusion (ACDF) is still considered the gold-standard surgical option in the treatment of cervical disc disease when conservative therapy fails .^[1,2]^ Since its description in the 1950s,^[3,4]^ many procedures including use of allograft bone and anterior plating, polyetheretherketone (PEEK) cages with anterior plating, and other interbody fusion devices have been reported .^[5]^

The ACDF treats the potentially debilitating effects of cervical degenerative disc disease (DDD) by providing long-term stabilization, maintaining disc space height and decompressing the neural elements.^[1,6–8]^ Supplementary fixation by an anterior cervical plate may be added to stabilize the segment, improve outcomes, and reduce the risk of pseudarthrosis .^[1,9]^ However, the use of anterior plating with its inherent prominence is associated with complications such as dysphagia.^[10–13]^

To avoid the drawbacks of plating or posterior instrumentation, zero-profile anchored cage systems have been designed for stand-alone fusion.^[14,15]^ The purpose of this study was to assess efficacy and safety of ACDF with a cervical interbody cage with integrated fixation (ROI-C, Zimmer Biomet, Troyes, France) at 2 years following surgery.

## 2 Materials and methods

### 2.1 Study design

This was a prospective multi-center study, conducted in France, of patients who underwent single- or multi-level ACDF with ROI-C cages.^[16]^ Between February 2010 and September 2015, 90 consecutive patients suffering from degenerative disc disease and/or moderate intervertebral instability were enrolled. Each investigator performed 3 surgeries before inclusion of his or her 1st patient. Inclusion criteria were:

- 18 years of age or older,
- No prior cervical spine surgery,
- Cervical degenerative disc disease, or intervertebral instability of discogenic origin with decreased segmental lordosis:
  - Single or multi-level,
  - Confirmed by imaging (radiography + magnetic resonance imaging [MRI] and possibly computed tomography [CT]),
  - Responsible for root and/or spinal cord symptoms,
  - Resistant to properly conducted conservative treatment.
- Patients who completed a self-administered questionnaire preoperatively, and who had preoperative MRI and static and dynamic radiographs of the cervical spine,
- Patients reimbursed by a social security system.

Exclusion criteria were:

- Multi-stage disease requiring the use of different implants on different segments to be treated,
- Hyper-mobility in flexion/extension, dislocation, hyper-rotation,
- Major degenerative or traumatic instability,
- Cervical canal narrowing requiring posterior decompression associated with anterior fusion,
- History of posterior instrumentation at the level concerned,
- Chronic, progressive rheumatic disease (rheumatoid arthritis),
- Metabolic bone disease: major osteoporosis, severe osteopenia, osteochondrosis,
- Systemic, spinal or localized infection, acute or chronic fever,
- Known allergy to the implant materials used,
- Tumor,
- Lack of compliance from the patient (non-cooperative. psychological instability, personal circumstances impeding the study follow-up),
- Smoking and context of work-related injuries were not exclusion criteria.

According to French regulation, each patient was appropriately informed of his/her rights regarding medical data collection and freedom to decline participation in the study.

### 2.2 Outcomes

Each patient was followed-up prospectively with preoperative and post-operative evaluations for 2 years. The primary outcome was fusion rate evaluated from CT-images by an independent laboratory at 1 and 2 years (if there was any doubt about fusion or CT image not obtained at 1-year follow-up). The quality of fusion was classified as follows:

- Certain fusion: bone continuity between the 2 vertebrae on at least 1 image in both sagittal and frontal planes.
- Doubtful fusion: bone continuity between the 2 vertebrae on at least 1 image in only 1 of the 2 planes of the CT.
- Fusion failure (pseudarthrosis): no bone continuity between the 2 vertebrae in either plane.

Clinical scores were evaluated pre-operatively, at 2, 6, 12, and 24 months and included visual analog scale (VAS) for neck and arm pain (0–100 mm), the Neck Disability Index (NDI 0-100%), and the short-form 36 (SF-36) quality-of-life questionnaire. Patients were also asked to rate their satisfaction (very satisfied/satisfied/not satisfied/dissatisfied) at 12 and 24 months.

Medication consumption (analgesic use, no medication, medication for other pathology) and professional status (working, sick leave, retired active, and retired inactive) was tracked during pre- and post-operative visits. All clinical complications were reported.

**Figure 1:**
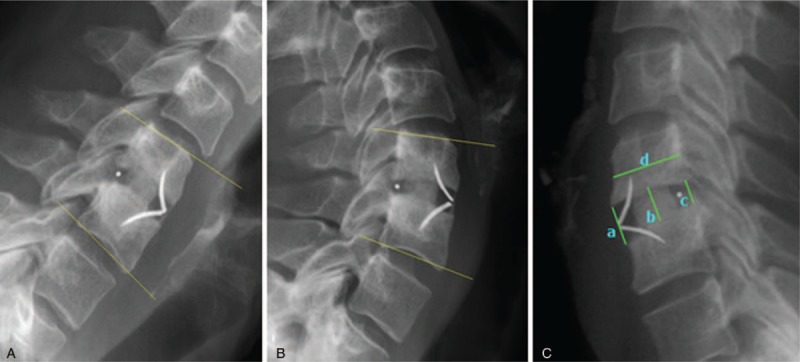
(**A**) Functional spinal unit alignment in flexion. (**B**) Functional spinal unit alignment in extension. (**C**) Radiographic measurements of disc height: (a) Anterior disc height, (b) Middle disc height, (c) Posterior disc height, (d) Sagittal diameter of the overlying vertebral body. Disc height index = [(a + b + c)/3]/d.

In addition to fusion assessment, radiological performance evaluations included overall cervical lordosis, and functional spine unit (FSU) lordosis, disc height index (DHI) and range of motion (ROM) at index FSU and adjacent levels. The DHI was measured using Inoue's method ^[17]^ from standing lateral radiographs (Fig. 1C). The ROM at index FSU (Fig. 1A–B) and adjacent levels were measured pre-operatively, at 2, 12, and 24 months from lateral radiographic images in maximum flexion/extension. Cervical lordosis and FSU lordosis were measured on radiographic images in standing neutral lateral positions pre-operatively, right after surgery, at 6 and 12 months. The ROM and lordotic angles were measured using Surgimap Spine software (Surgimap, version 2.2.9.9.8).

### 2.3 Ethics statement

The protocol was registered and received approval from the French Advisory Committee on Data Processing Related with Health Research (CCTIRS) and National Commission for Data Protection and Liberties (CNIL). According to the French regulation, each patient was appropriately informed of his freely participation to the study and of his rights towards medical data collection. Observational study does not modify the surgeon-patient relationship or the usual care of patients. No act or particular examination being requested which are not used in current practice. No visit was imposed. Graft choice was determined by investigator.

### 2.4 Statistical analysis

All available data have been taken into account. The Wilcoxon matched-pairs signed rank test was used for comparisons between preoperative and post-operative continuous data. The McNemar's test was used for comparison of categorical data. The significance level was *P* < .05. Statistical analyses were conducted using the statistical program R (version 3.3.2; https://www.R-project.org).

## 3 Results

A total of 90 patients included in this study received 125 ROI-C devices. Sixty-two patients (68.9%) were treated at one level. The mean surgery duration was 84.5 minutes (range, 35–180). The mean hospital duration was 3.3 days (range, 0–9). Table 1 summarizes demographic, preoperative clinical and surgery data.

**Table 1:**
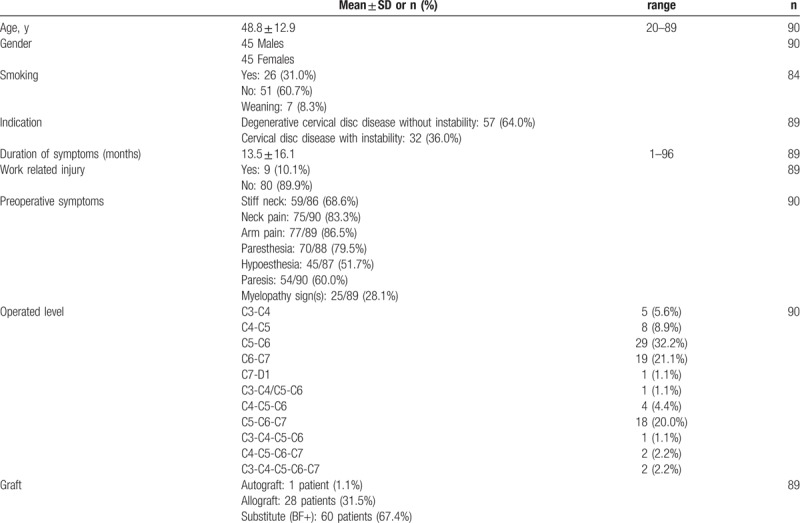
Demographic and preoperative clinical data of the study population.

At 2 years, 91/125 CT's (66 patients) were available for fusion assessment. Results indicate 85/91 levels were fused, 5/91 levels were doubtful and 1/91 failed to achieve fusion. Thus, the fusion rate was 93.4% (95% confidence interval [CI] ranging from 86.2% to 97.5%). Six levels were evaluated as doubtful fusion or failure. The 4 concerned patients were women ages 37 to 79 years who received an allograft. At least 3 of these 4 patients were not smokers (the information was missing for the 4th), and 3 were implanted for multi-level disc disease and/or instability, only 1 for a single-level indication (instability).

**Figure 2:**
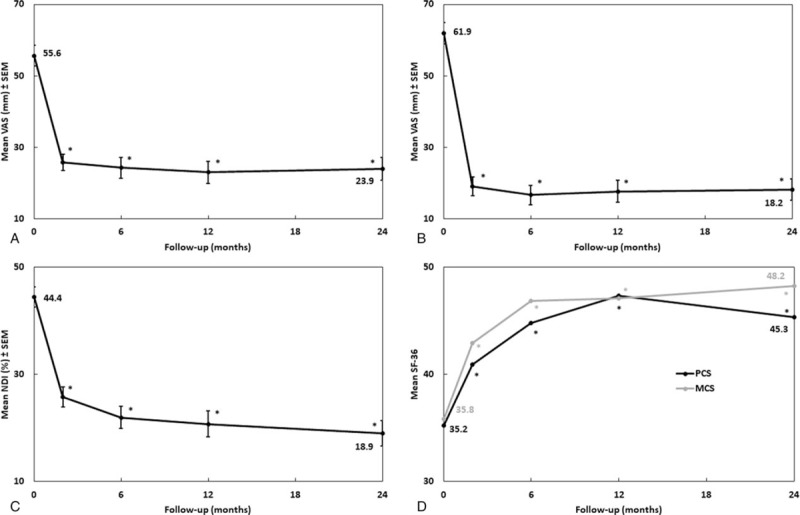
Clinical outcomes over follow-up. Results are expressed as mean ± SEM. ∗*P* ≤ .05 compared to preoperative baseline: (**A**) visual analog scale (VAS 0–100 mm) for neck pain. (**B**) visual analog scale (VAS 0–100 mm) for arm pain. (C) Neck disability index (NDI 0–100%). (D) SF-36 score (PCS = physical composite score; MCS = mental composite score).

At 24-month follow-up, all clinical scores were significantly improved compared to baseline (Fig. 2). The NDI score significantly improved at 2 months and improved by an average of 26.6% at 24-month follow-up compared to baseline (Fig. 2C). Both arm and neck pain decreased immediately after surgery. The difference from baseline was statistically significant from 2 months and throughout the follow-up period (Fig. 2A, B). Outcomes for quality of life also show improvement for both mental and physical composite scores (Fig. 2D).

**Figure 3:**
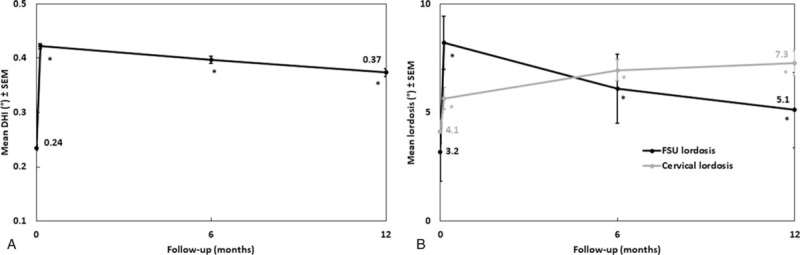
Lordosis over follow-up. Results are expressed as mean ± SEM. ∗*P* ≤ .05 compared to preoperative baseline: (**A**) Disc height index (DHI). (**B**) Cervical and FSU lordosis. DHI = disc height index, FSU = functional spine unit.

**Figure 4:**
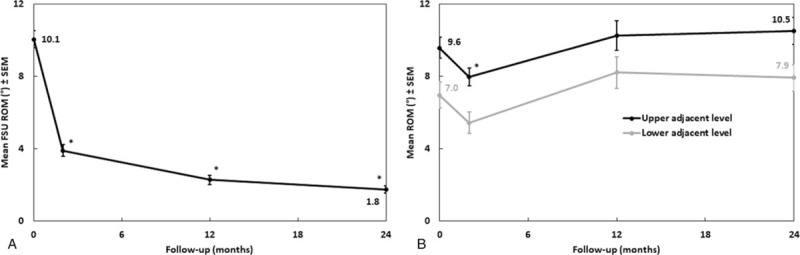
Mobility over follow-up. Results are expressed as mean ± SEM. ∗*P* ≤ .05 compared to preoperative baseline: (**A**) ROM at the index FSU. (**B**) ROM at adjacent levels. FSU = functional spine unit, ROM = range of motion.

One patient was reoperated for symptomatic pseudarthrosis, and 3 were reoperated for adjacent segment disease. There was only 1 case of dysphagia, which resolved in less than 12 months. Other postoperative complications included 1 neuro-motor deficit, 5 anchor fractures (5/249), and 5 (5/125) cases of without clinical consequences or reoperation (Table 2). The DHI and both cervical and FSU lordosis increased significantly after surgery (Fig. 3). Mean FSU ROM decreased significantly from 10.1° preoperatively to 1.8° after 2 years (Fig. 4A). Proximal and distal adjacent discs showed no significant change of motion compared to baseline (Fig. 4B).

**Table 2:**
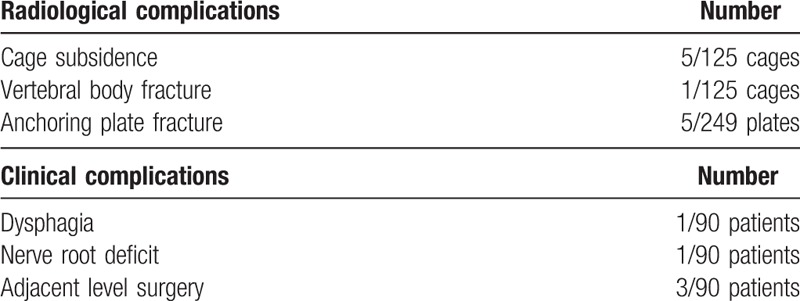
Radiological and clinical complications.

The rate of patients using analgesics decreased significantly from 79.5% before surgery to 13.3% at 24 months. Professional status was significantly upgraded with an increase of working patients (from 27.6% preoperatively to 54.4% at 2 years) and decrease in the percentage of patients on sick leave. At 24-month FU, clinical results appreciation of each patient in the study population by the surgeon-investigator was poor in none, average in 15.3%, good in 35.6% and excellent in 49.2%. Additionally, 98.4% of patients were satisfied with overall surgery results.

## 4 Discussion

The ACDF with plate and screws is a standard procedure .^[18,19]^ It can increase fusion rates, maintain or improve cervical sagittal alignment and primary and secondary stability. However, ACDF with plate and screws is associated with complications that include screw or plate dislodgement, soft tissue injury, dysphagia and adjacent segment disease.^[20–23]^ The present study was designed to evaluate the safety and efficiency and assess both clinical and radiological outcomes of a cervical interbody cage with integrated fixation. The results demonstrated that clinical scores improved significantly compared to baseline at all-time points after surgery until final FU. A high interbody fusion rate and a low complications rate were also observed.

Pseudarthrosis after ACDF is not rare. Although pseudarthrosis is not always symptomatic, bone nonunion has been linked to poor clinical outcomes.^[24,25]^ Fraser et al reported, in a meta-analysis of 25 studies with 2682 patients overall, fusion rates of 92.1%, 79.9%, and 65.0% for 1-level, 2-level, and 3-level ACDF, respectively .^[26]^ In the present study the fusion rate at 2 years was 93.4%. Other studies investigating the same device reported similar high rates of bony fusion ranging between 95.2% and 100%.^[27–29]^ It should be noted that 31% of the present patients were smokers. Although smoking has been shown to negatively affect fusion rates in patients undergoing fusions of the cervical and lumbar spine,^[30,31]^ in the present study and in another on single-level ACDF ^[32]^ smoking had no observable impact on fusion rate. In the present study, 1 patient operated at a single level had revision surgery (autograft with plate fixation) for symptomatic pseudarthrosis. In 91 operated levels among the patients examined by CT 2 years after ACDF, there was 1 definite and 5 possible non-unions, but they were not symptomatic and did not require additional surgery. As regards the type of graft material used in ACDF, there is great variability across the world.^[6]^ It has been found in a systematic review that there is no significant difference in fusion rates or patient outcomes when utilizing autologous graft or allograft .^[7]^ In the present study, most patients (67.4%) had a bone graft substitute; all 4 of the patients with possible or certain non-fusion had been operated with allograft. Other factors, then smoking, that increase the risk of pseudarthrosis are malnutrition, obesity, osteoporosis, diabetes, and rheumatoid arthritis.^[33]^ People who use oral steroids or non-steroidal anti-inflammatory medications are also at higher risk. The elderly are more likely to develop pseudarthrosis as well as those who do not allow enough recovery time following fusion surgery. Too much activity will prevent the bones from fusing properly. In our study, there is no particular risk factor that emerges.

In accordance with other clinical series,^[1,9,10,21]^ the present results at 2 years after ACDF demonstrated statistically significant improvement in NDI, VAS for arm and neck pain, and SF-36 physical component score (PCS), and mental component score (MCS) results associated with high rates of patient satisfaction. Similar observations have been reported in a number of studies evaluating the clinical efficacy of ACDF using cages with integrated fixation.^[10,11,13,16,27,29]^ Dysphagia is a relatively common complication after conventional ACDF with an anterior plate.^[9–11,14,28,29,34,35]^ Only 1 patient (1.1%) experienced dysphagia, which resolved less than 12 months after the surgery. In our study, we could not determine the reason of dysphagia. The causes of dysphagia after ACDF are multifactorial and involve neuronal, muscular, and mucosal structures. Some possible ways include over retraction of the esophagus and other soft tissues, any type of obstruction that pushes up against a structure involved in swallowing, such as the esophagus or a nerve, and accidentally nicking a structure, such as the esophagus or a nerve.^[36]^ A dysphagia rate of 1.1% is very low compared to plate-augmented ACDF in the literature.^[16,35]^ The results thus obtained are compatible with the results of a meta-analysis of cohort studies showing that zero-profile anchored cages had a lower risk of postoperative dysphagia than cages with anterior plate fixation after ACDF, 1.0% vs 10.8% at 12 months and 0.8% vs 4.2% at 24 months after surgery.^[14]^ However, subsidence is one of the major concerns about cages without plate fixation. Due to several factors such as bone quality and surgical method, rates of subsidence after ACDF vary from 5.4% to 55.6%.^[37]^ The present study reported a subsidence rate of 4%, while Bucci et al ^[16]^ indicated that no subsidence occurred in a retrospective study of 110 patients with the present implant.

With regards to radiological results, the mean FSU ROM was below 2° at 2 years reflecting the high fusion rate, including a mean of 3.6° in patients with doubtful or failed fusion. The DHI and cervical and FSU lordosis of all patients were improved significantly after surgery at all-time points. Despite not being able to compare the data because of different measurement methods, other studies have also reported obvious improvements compared with the preoperative status.^[35,38,39]^ The ACDF with anterior plating is also thought by many to hasten degenerative changes in adjacent segments, but it is open to debate as to whether incidence of adjacent segment degeneration is related to natural degeneration or increased biomechanical stress resulting from adjacent fusion.^[21,35,40,41]^ While Bucci et al reported, in a study of 110 patients, 1 sub-surgery for adjacent segment (0.9%) [16], Hofstetter et al reported a rate of 5.7% [27], both using the present device. The present study showed 3.3% of adjacent level surgery up to 2 years after ACDF. This is consistent with a meta-analysis of randomized controlled trials, in which Dong et al indicate that adjacent segment reoperation in ACDF patients ranged between 0.9% and 11.1%.^[42]^

It should also be noted that medication consumption declined significantly and significant resumption of work was observed at 2-year FU. Similarly, the present patients have maintained a high level of satisfaction 2 years after surgery consistent with the high clinical results assessment by the investigating surgeons. Comparable data are rare in the literature and influenced by age, disability claims and workers compensation even though patients treated with arthroplasty have been shown to return to work sooner than ACDF patients .^[43]^

The main limitation of the present study is a potential selection bias arising from the fact that the study was not randomized. However, the present report may contribute to the evaluation of everyday clinical practice by providing real-world evidence. Indeed, smoking and work-related injuries were not exclusion criteria. A systematic review comparing non-randomized observational studies and randomized controlled trials concluded that both types of study designs can achieve similar results.^[44]^ Going even further, Grob et al recommend against discrediting observational studies as a relevant source of evidence in spine surgery.^[45]^

## 5 Conclusion

The results of the present study investigating the clinical and radiological outcomes of ACDF using a cervical interbody cage with integrated fixation showed that this system is safe and effective for the treatment of cervical degenerative disc disease. Substantial improvement in clinical scores and low incidences of postoperative dysphagia and subsidence were obtained by this straight-forward procedure.

## Author contributions

**Conceptualization:** Nicolas Lonjon, Emmanuel Favreul, Jean Huppert.

**Data curation:** Nicolas Lonjon, Emmanuel Favreul, Jean Huppert, Eric Lioret, Manuel Delhaye.

**Formal analysis:** Ramzi Mraidi.

**Funding acquisition:** Nicolas Lonjon, Jean Huppert.

**Investigation:** Nicolas Lonjon, Emmanuel Favreul, Jean Huppert, Eric Lioret, Manuel Delhaye.

**Methodology:** Nicolas Lonjon, Jean Huppert.

**Software:** Ramzi Mraidi.

**Supervision:** Nicolas Lonjon, Jean Huppert.

**Validation:** Nicolas Lonjon, Emmanuel Favreul, Jean Huppert, Eric Lioret, Manuel Delhaye.

**Visualization:** Ramzi Mraidi.

**Writing – original draft:** Nicolas Lonjon, Ramzi Mraidi.

**Writing – review & editing:** Nicolas Lonjon, Emmanuel Favreul, Jean Huppert, Eric Lioret, Manuel Delhaye, Ramzi Mraidi.
